# Prostate specific membrane antigen binding radiopharmaceuticals: Current data and new concepts

**DOI:** 10.3389/fmed.2022.1060922

**Published:** 2022-12-06

**Authors:** Oliver Sartor, Ali Baghian

**Affiliations:** ^1^Department of Medicine, Tulane University School of Medicine, New Orleans, LA, United States; ^2^Department of Urology, Tulane University School of Medicine, New Orleans, LA, United States; ^3^Section of Hematology and Medical Oncology, Deming Department of Medicine, Tulane University School of Medicine, New Orleans, LA, United States; ^4^Department of Population and Public Health Sciences, University of Southern California, Los Angeles, CA, United States

**Keywords:** PSMA, prostate cancer, actinium-225, alpha particles, targeted alpha therapy, clinical trials

## Abstract

Prostate specific membrane antigen (PSMA) represents a validated target for prostate cancer therapeutics. The phase III VISION study with ^177^lutetium (^177^Lu)-PSMA-617 represented a pivotal step forward and the FDA has now approved this agent in advanced metastatic castrate-resistant prostate cancer (mCRPC). A number of other PSMA targeted radiopharmaceuticals are now under development. Some of these agents are targeted to PSMA via monoclonal antibodies such as J591 and TLX591. Others are targeted to PSMA via small molecules such as PSMA-617, PSMA I&T, MIP-1095, etc. In addition to the use of various ligands, multiple isotopes are now in clinical trials. Beta emitters in development include ^177^Lu, ^131^iodide (^131^I), and ^67^copper (^67^Cu). Targeted alpha emitters potentially include ^225^actinium (^225^Ac), ^227^thorium (^227^Th), and ^212^lead (^212^Pb). Phase III trials are underway with both ^177^Lu-PSMA-617 and ^177^Lu-PSMA I&T in mCRPC. Single dose phase I trials are complete with ^225^Ac-J591 but additional data are need to launch a phase III. Data are promising with ^225^Ac-PSMA-617 but concerns remain over salivary and renal toxicity. Tandem therapies are also considered combining both beta and alpha-targeted therapy. Taken together the field of PSMA targeted radiopharmaceuticals is rapidly developing. The targeted alpha therapies are particularly promising and several developmental paths forward are being considered in the near future.

## Introduction

Prostate cancer is a common disease with a preponderance of bone metastases when spread is apparent. As such, it has long been targeted with bone-targeted radiopharmaceuticals including the beta-emitters ^32^phosphorus, ^89^strontium, ^153^samarium lexidronam, and the alpha-emitter ^223^radium (^223^Ra) (1–4). Palliative effects on bone pain have been documented for each of these isotopes in various studies. ^223^Ra, which was the first alpha particle to be FDA approved, after demonstrating overall survival improvements in a randomized phase III study. This finding was catalytic for interest and investment in the broad field of therapeutic radiopharmaceuticals (4).

More recently prostate specific membrane antigen (PSMA) targeted therapies have become more prominent. A small background is justified to cover this space from a conceptual perspective. All the PSMA targeted agents in therapeutic trials to date can be readily classified in terms of small molecules or antibodies. The small molecules typically have four components, a PSMA binding moiety, a linker, a chelator, and an isotope (5–7). All the antibodies in current development are targeted to bind the extracellular domain of PSMA (at a different site than the small molecules). All the antibodies also use a chelator/isotope combination. Despite the fact that chelators and linkers are key components of the targeted therapeutics, the chemistry of those components are not covered in detail here as that is beyond the scope of this manuscript.

The PSMA small molecules typically have a Glu-ureido component that serves as the PSMA binding motif. The linker is a key component that affects tumor targeting, pharmacokinetics, and cellular uptake. Interestingly, various linker/chelator moieties have quite distinct internalization ratios (5–7). Thus the combination of linkers and chelators are critical to the PSMA small molecules and must be carefully considered in design considerations. Historically MIP-1095, PSMA-617, and PSMA I&T are three key small molecules that have been studied more than others (8–10). The most commonly studied antibody has been J591 (11). A structurally modified J591 (TLX592) and another anti-PSMA monoclonal antibody have recently entered the clinic as well (see NCT04726033 and NCT03724747, respectively).

Prostate specific membrane antigen expression is a key determinant of anti-tumor efficacy though debate continues regarding optimal selection of patients (12). PSMA is expressed in the vast majority of prostate cancer patients though heterogeneity clearly exists. Upregulation of PSMA expression in cancers is typical, relative to prostate tissue. In addition to prostate cancer and prostate tissue, PSMA expression is also encountered in neovasculature, proximal renal tubules, central nervous system, salivary tissue, and in the duodenal/jejunal brush border (13–15). Some data suggest that PSMA may be upregulated by hormonal inhibition using agents such as enzalutamide or abiraterone (16, 17). The clinical consequences of this hormonally induced PSMA upregulation are unclear at this time but potential synergy has been suggested between PSMA targeted therapeutics and potent androgen signaling inhibitors.

Prostate specific membrane antigen expression in the tumors can be assessed by PSMA PET imaging which gives quantitative uptake information, especially if software support is used. Much information is now available on PSMA PET and multiple reviews are published (18). The ratio between tumor uptake and benign tissue uptake is critical information that can be assessed and used to predict (to some degree) anti-tumor efficacy. Though heterogeneity is a key hallmark of cancer, and PSMA heterogeneous expression is well documented, the use of radionuclides helps to overcome the problem of heterogeneity by radiating both the cell to which it binds, and also the surrounding tumor microenvironment. One of the more commonly utilized isotopes ^177^Lutetium (^177^Lu), has a maximum and mean path length of the beta particle of about 1.7 and 0.23 mm (respectively) in soft tissue (19). Thus, deposition of the isotope on a PSMA expressing tumor cell can be expected to provide radiation to surrounding tissues adjacent to the area of isotopic deposition. Alpha particles have a much shorter range and though estimates vary, typical alpha particles have a path length of less than 100 microns (20). Given the mass of alphas, relative to betas, the linear energy transfer, and the degree of induced DNA damage of alpha particles, far exceed that of the beta particles (21). Regardless, the penumbra of radiation around the site of deposition is a key concept underlying the mechanism of action for this class of therapeutic.

## Phase III trials completed with ^177^Lu-PSMA targeted agents

The PSMA targeted beta emitter ^177^Lu-PSMA-617 was tested in the PHASE III VISION trial (22). No other phase III trials have been reported to date with PSMA targeted radiopharmaceuticals. The VISION study demonstrated that the PSMA targeted isotopic therapy prolonged survival in heavily pretreated patients with metastatic castrate-resistant prostate cancer (mCRPC) and this agent is now FDA approved for PSMA PET positive men with progressive disease after prior treatments with androgen deprivation therapy (ADT), newer androgen-axis pathway inhibitors (ARPIs, i.e., abiraterone, enzalutamide, darolutamide, and apalutamide) and at least one taxane-based chemotherapy (typically docetaxel).

The phase III VISION trial selected patients by ^68^Ga-PSMA PET scan criteria. All patients had to have a PSMA PET positive (uptake > liver parenchyma) metastatic lesion. No tumor lesion (≥1 cm) could be PSMA negative (uptake < liver) in a visceral organ or a lytic bone lesion. No PSMA negative tumor lesion could be ≥2.5 cm in a lymph node. The negative selection criteria are important (23). Data from the VISION trial demonstrated responses as measured by both PSA decreases and reduction in tumor size. Time to radiographic progression was substantially improved as well. Interested readers are referred to the original VISION manuscript for further reading on this phase III trial (22).

It is important to understand what was not learned in the VISION trial, as well as what was learned. The optimal dose for ^177^Lu-PSMA-617 is still not known. There is some belief that dosing remains sub-optimal and formal phase I studies with this agent have never demonstrated a dose limiting toxicity. The optimal selection of patients is still not clear and imaging as a predictive biomarker is far from perfect (24). The role of the standard of care therapies is still not clear, but those that received a combination of ARPIs such as enzalutamide or abiraterone had a somewhat better survival as compared to those that did not (22). There was no retreatment allowed in the VISION study. Once treatment was stopped it could not be restarted for relapse at a later date. No PSMA PET imaging was used in VISION after therapy was started and the relationship between PSMA PET changes after treatment initiation are not ascertained. Clearly there is much more to learn about PSMA targeted ^177^Lu therapies.

## Phase III trials underway with ^177^Lu-PSMA targeted isotopic therapy

Several immediate strategies are evident for ^177^Lu-PSMA-617 and phase III trials are now ongoing with earlier stage prostate cancer patients. In the patient with mCRPC ^177^Lu-PSMA-617 is being tested in men with progression post-ARPI without prior taxanes. This trial (PSMAFore) (see NCT04689828) is currently in process and evaluates rPFS as the primary endpoint. For men with metastatic castrate-sensitive prostate cancer (mCSPC), ^177^Lu-PSMA-617 is being evaluated in a phase III trial termed PSMAddition (NCT04720157). This trial uses an ADT + ARPI ± ^177^Lu-PSMA-617 design and incorporates an rPFS endpoint. Both PSMAfore and PSMAddition utilize the same dose of ^177^Lu as that in the VISION trial (7.4 GBq per dose q 6 weeks, up to a maximum of 6 doses).

SPLASH (NCT04647526) sponsored by POINT Biopharma and ECLIPSE (NCT05204927) sponsored by Curium are both phase III trials and both use a design similar to PSMAfore. The patient population is mCRPC patients with progression post-ADT/ARPI. Patients are required to be PSMA PET positive and rPFS is the primary endpoint. Both trials are currently accruing patients. Both the SPLASH and ECLIPSE trials use an alternative PSMA targeting agent, PSMA I&T (also termed ^177^Lu-PNT2002 by POINT Biopharma). Dosing in the SPLASH trial is lower than for ^177^Lu-PSMA-617. In SPLASH the doses are 6.8 GBq every 8 weeks up to a maximum of 4 doses. For ECLIPSE, the dose of 7.4 GBq is given every 8 weeks for up to 4 doses. ^177^Lu-PSMA I&T on a per dose basis, may cause more renal radiation as compared to ^177^Lu-PSMA-617 (25).

Given regulatory concerns, renal dose limitations of 23 Gy have generally been used in dose planning for the radiopharmaceuticals, despite the fact that this dose limitation was based on external beam studies and likely is not appropriate to apply to systemic radiopharmaceuticals (26). Regardless, legitimate issues regarding late renal toxicity exist, especially regarding the use of alpha particles. Long term safety issues are a concern for regulators and clinicians alike. Long term survival is more of an issue for earlier stage patients than patients with advanced disease who are progressing after multiple lines of therapy.

## Other prostate specific membrane antigen targeted isotopes: Antibodies, small molecules, and albumin-binders

Isotopes can be targeted to PSMA via small molecules, antibodies, and more. Most of the initial work has focused on PSMA-617 and PSMA I&T but multiple other molecules are under development. Each molecules has potential merits and in the end, only careful clinical trials will distinguish those that are best. Of note, antibodies bind to a distinct aspect of the PSMA molecule as compared to small molecules which target the PSMA “binding pocket.” One potential way to mitigate renal issues with small molecules is to use albumin binding as a way to diminish glomerular filtration.

The first PSMA targeting agent using in human trials was MIP-1095 using ^131^I as the isotope (27). The initial trials with ^131^I-MIP-1095 were clearly positive as measured by PSA declines and even though more trials are in process, the lack of planned phase III trials likely means that MIP-1095 will not move forward as a practice-changing therapy.

An antibody to PSMA (J591) which has been studied extensively in men with mCRPC using both beta and alpha emitters. Phase II studies with ^177^Lu-J591 indicate that the antibody is associated with some provocative long term survival data (28) but these data were older and collected in an era without many of the effective therapies commonly utilized today. Data with ^177^Lu-J591 indicate that this agent has significant marrow suppression especially thrombocytopenia (28). The PSA response rate with ^177^Lu-J591 is relatively low compared to small molecules and the data on radiographic responses/progression are quite limited. Though overall survival (OS) is the gold standard for activity of an agent, the relatively sparse un-randomized data sets currently available make conclusions about the activity of this agent somewhat problematic. A phase III study (PROSTACT) of ^177^Lu-J591 is planned in the mCRPC space post-ARPI but this trial has yet to start accrual (NCT04876651).

Various PSMA targeting agents are under investigation and these are summarized in Table 1. A PSMA antibody is in phase I trials with Bayer using ^227^Th as an isotope (NCT03724747). ^227^Th has half-life of 18.7 days and decays to ^223^Ra which in turn has a half-life of 11.4 days. It is unlikely that cellular retention will last as long as the half-lives of ^227^Th and the daughters. No data are yet reported on NCT03724747 but the trial is no longer accruing patients. Additional PSMA targeting antibodies (TLX592) are in development. TLX592 is a modified J591 with more rapid blood clearance as compared to J591. It is anticipated this TLX592 will be developed with ^225^Ac. Current studies with TLX592 are examining ^64^Cu based imaging (NCT04726033) to better understand the distribution and binding of this antibody. Future studies with alpha-emitters are planned with TLX592. Once imaging studies are done, the antibody-isotope conjugate can be readily adapted for therapy.

**TABLE 1:** Synopsis of prostate specific membrane antigen (PSMA) targeted molecules in current development or consideration.

	Sponsor	Beta	Alpha	Prospective data published	Beta trial	Alpha trial	NCT number
**Monoclonal antibodies**							
J591	Cornell	^177^Lu		I/II			Multiple
J591	Cornell		^225^Ac	I			NCT03276572
TLX591	Telix	^177^Lu			I		NCT04786847
PSMA TTC	Bayer		^227^Th			I	NCT03724747
**Small molecules**							
MIP-1095	Lantheus	^131^I			II		NCT03939689
PSMA I&T	POINT	^177^Lu			III		NCT04647526
PSMA I&T	Curium	^177^Lu			III		NCT05204927
PSMA-617	Novartis	^177^Lu		II/III	III		Multiple
PSMA-R2	Novartis	^177^Lu			I		NCT03490838
SAR-bis-PSMA	Clarity	^67^Cu			I		NCT0486860
EB-PSMA-617	Peking Union	^177^Lu		I			NCT03403595
NG-001	Nucligen		^212^Pb				Planned
PNT-2001	Point		^225^Ac				Planned
PSMA-617	Novartis		^225^Ac			I	NCT04597411
PSMA I&T	Excel diagnostics		^225^Ac			I	NCT05219500

Small molecules in development include a ^67^Cu-PSMA binding agent by Clarity Pharmaceuticals. This agent is being developed as a theranostics pair with ^64^Cu as a PET imaging agent (NCT04868604). Nucligen, a small Norwegian company, is developing a ^212^Pb-PSMA binding molecule NG001. The NG001 strategy may involve a dual approach using both ^224^Ra and ^212^Pb (29). ^224^Ra will target bone similar to ^223^Ra. Human trials are yet to be announced. The PSMA binding small molecule “R2” has been in phase I trials using ^177^Lu as an isotope but accrual was terminated (NCT03490838). No results have been reported. PSMA targeting compounds binding ^211^At are described (30) but are yet to be used in the clinic.

Noria developed a small molecule that binds albumin as well as PSMA and this molecule, or a similar one, will likely be developed by Bayer (31). Other albumin binding PSMA targeted molecule are in development, some including ibuprofen conjugated linkers (32). Albumin binding molecules such as Evans Blue modified PSMA-617 (EB-PSMA-617) are also under development (33) and slated for additional human clinical trials (NCT04996602). Albumin binding may or may not be effective in diminishing salivary and renal uptake but some data indicate that tumor retention can be improved relative to renal/salivary uptake (31, 34). The key elements will be the ratio of binding to target and non-target tissues, the degree of isotopic retention in the tumor, and the salivary/renal dosimetry. Human therapeutic data are not yet reported for these agents.

## Prostate specific membrane antigen targeted alpha particles in current clinical trials: Monoclonals, small molecules, and multiple isotopes

The phase III ALSYMPCA trial demonstrated an OS benefit with ^223^Ra in bone predominate mCRPC (4) but there is only one published prospective phase I alpha emitter trial using PSMA targeting. There is no need to cover ^223^Ra here given multiple reviews have been done, but the promise of targeted alpha therapy in prostate cancer is large and deserves mention.

The PSMA targeted monoclonal antibody J591 conjugated to ^225^Ac (^225^Ac-J591) has been tested in a small phase I single dose escalation trial (NCT03276572) with no PSMA PET selection criteria (35). No dose limiting toxicities (DLTs) were observed in this trial. The maximum dose tested was 93 kBq/kg as a single dose. Patients as a whole were heavily pretreated, including pretreatments with ^177^Lu-PSMA for a number of patients. Thrombocytopenia and nausea was somewhat problematic and some patients had excessive fatigue but as stated, no DLTs were observed. PSA declines were seen in the majority of patients. Followup was short and incomplete. Radiographic progression-free survival was not reported. Future studies are planned using more than one dose of ^225^Ac-J591 (NCT04506567). Clearly more work is needed before a phase III can be launched. A Phase I/II Trial of pembrolizumab and androgen-receptor pathway inhibitor with or without ^225^Ac-J591 is planned in mCRPC (NCT04946370). Additional phase I/II trials are planned with ^225^Ac-J591 + ^177^Lu-PSMA I&T (NCT04886986).

^227^Th-PSMA TTC monoclonal antibodies are also in formal phase I trials now (NCT03724747) but the trial is no longer accruing. As noted, the half-life of ^227^Th (an alpha emitter) is likely too long for optimal dose delivery. ^225^Ac-PSMA-617 is now in a formal phase I trial in South Africa and Australia (NCT04597411). Phase I trials are eventually planned with ^225^Ac-TLX592 and others. Clearly the activity of targeted alphas are noteworthy. Optimal dose and schedules are not yet established.

No formal phase I study has been reported for small molecules such as PSMA-617 or PSMA I&T with an alpha emitting payload. In an important study from Heidelberg with PSMA-617 reporting on 14 total patients looking at dose escalation (36), salivary gland toxicity (xerostomia) was reported as being dose limiting however only 2 patients reported a grade 2 xerostomia. Doses above 100 kBq/kg were deemed unfeasible though a formal phase I assessment was not reported. Extreme dry eyes were also reported in a single patient treated at 200 kBq/kg. Myelosuppression and renal toxicity were not problematic in the short term but there are potential concerns in longer term followup. This experience from Heidelberg with ^225^Ac-PSMA-617 suggested that 100 kBq/kg every 8 weeks was a tolerable and effective dose with 2−4 doses being typically administered (36). There was no dose response noted in the small group of patients being treated with 100−200 kBq/kg. Some have endorsed the concept of decreasing the targeted alpha dose over time given the tumor/normal tissue ratio decreases with lower tumor burden.

Dose limiting toxicity can be controversial and assessed in different ways by different investigators. The definitions of intolerable treatment also depend to some extent on the underlying disease being treated and the physician’s experience at handling toxic therapies. Asymptomatic men with good prognosis are quite distinct from symptomatic men near death from an aggressive underlying cancer and what is tolerable in one setting may be deemed intolerable in another. Xerostomia, dry eyes, nausea, and fatigue are areas where considerable variation in tolerability may be encountered from patient to patient and what is tolerable for one may not be tolerable for another. Salivary gland toxicity resulting in xerostomia is an area of special concern with small molecule PSMA-binders (see below).

Dosimetry with alphas studies are problematic. There are multiple assumptions regarding the relative biologic efficacy, issues related to microdosimetry, flow rates in tubular spaces, the exact range of the emitted alpha, and the potential diffusion of daughters. Recoil from alpha emissions renders ^225^Ac daughters free from the chelate and these “free” isotopes may cause additional off-target damage in the patient. In the ^225^Ac studies there is additional uncertainty regarding dosimetry because an unknown percentage of ^213^Bi (half-life 45.59 mins) likely leaves the targeted cell by diffusion and the ultimate whereabouts of this radionuclide is potentially problematic. Renal toxicity with ^213^Bi is a theoretical possibility (37). Tracing certain isotopic daughters is feasible using selective assessments of specific energies for photon emissions and novel strategies are evolving in the field of alpha dosimetry (38–40).

Three meta-analyses have been reported using ^225^Ac-PSMA-617 (41–43) and recent reviews with ^225^Ac-PSMA-617 have been presented as well (44). Thus, an exhaustive review of the literature is not warranted here. Suffice it to say that the PSMA targeted alpha therapy data to date are impressive with regard to PSA declines in heavily pre-treated patients including those pre-treated with ^177^Lu-PSMA targeted therapies. The durability of these responses are not yet clear.

^225^Ac-PSMA-617 has been used both in monotherapy and in tandem therapy with ^177^Lu-PSMA-617. For monotherapy in mCRPC chemotherapy naïve patients, individual doses were reported in the 8 MBq range in South Africa, followed by escalation or de-escalation depending on response to therapy (45). In a large German monotherapy ^225^Ac-PSMA-617 study from Heidelberg, the doses were initially 100 kBq/kg (46). In the monotherapy series from India, men were treated with ^225^Ac-PSMA-617 (100 kBq/kg) at 8 week intervals (47, 48). Four of the larger reported experiences are described in Table 2. Cohorts from all four trials exhibited widespread disease on PSMA imaging with median PSA ranging from 57 to 222 ng/ml. The majority of subjects in one of the series from India (47) were ECOG performance status 3 thus these results are not broadly representative of what might be expected in future prospective trials. The majority of patients in the South African series (45) were not pretreated with abiraterone/enzalutamide/taxanes. Results from the South African series are impressive but these data clearly represent patients with a better prognosis. Many of the patients in these four trials had previously been treated with radiopharmaceuticals including ^177^Lu-PSMA-617 as shown in Table 2.

**TABLE 2:** Synopsis of selected experiences with 225Ac-PSMA-617 treated prostate cancer patients.

			Percentage of patients pre-treated						
^225^Ac-PSMA-617 study	Number of patients	^225^Ac dose	Novel hormones (Abi/Enza)%	Docetaxel/ Cabazitaxel %	^177^Lu-PSMA-617/^223^Ra %	Median PSA at time of trial entry	ECOG > 2 (%)	PSA > 50% decline	Median OS months
Sathekge et al. (45)	73	8 MBq every 8 weeks^†^	2/0	51/0	14/0	57	2	70% (51/73)	18
Kratochwil et al. (46)	40	100 kBq/kg every 8 weeks	85/60	70/17.5	0/22.5	169	20	60% (24/38)	>12
Sen et al. (48)	38	100 kBq/kg every 8 weeks	63/34	100/11	24/5	147	0	66% (25/38)	12
Yadav et al. (47)	28	100 kBq/kg every 8 weeks	79/36	79/4	54/0	222	72	39% (11/28)	17

^†^Subjects were treated with 8 MBq then 7, 6, or 4 Mbq very 8 weeks on basis of response to treatment.

Abi, abiraterone; ECOG, Eastern Cooperative Oncology Group; Enza, enzalutamide; PSA, prostate specific antigen; OS, overall survival.

There is additional experience with ^225^Ac-PSMA I&T from retrospective German series. One experience with PSMA I&T used 6.0−8.5 MBq ^225^Ac-PSMA I&T in 14 patients for 1-5 cycles every (q) 8 weeks in mCRPC patients (mostly pretreated with ^177^Lu-PSMA targeting agents) (49). Half the patients had a PSA decline ≥50%, 5/11 of the patients pretreated with ^177^Lu-PSMA had a PSA decline of ≥50%. New onset grade I/II xerostomia was noted in 5/14 and this concern is addressed below in more detail A phase II with ^225^Ac-PSMA I&T q 8 weeks is now underway (NCT05219500) using 100 kBq/kg for dose 1, followed by dose de-escalation in responding patients (using investigator discretion). A phase I using ^177^Lu-PSMA I&T in combination with ^225^Ac-J591 is also planned (NCT 04886986). A single case report has been reported with treatment consisting of two cycles of ^213^Bi-PSMA-617 with a cumulative activity of 592 MBq (50).

Though formal phase 1 studies are not performed, several investigative groups are planning to perform initial studies using ^212^Pb-based PSMA ligands. The shorter half-life and single alpha emission is of interest.

## Late renal toxicity concerns and salivary concerns: Areas of special interest

Xerostomia from salivary toxicity and bone marrow suppression are issues that have received considerable attention but the issue of late renal toxicity is a potential special concern following targeted alpha therapy. Data from neuroendocrine tumors indicate that late renal toxicity following treatment with ^225^Ac-labeled small molecules (DOTATOC) can be a problematic issue for some patients (51). The renal toxicity rarely manifested in less than a year and could be delayed 2−4 years. This cautionary note is worthwhile to consider and the short term issue surrounding alpha success needs to be tempered with a concern over late effects that may be deleterious. Those with a short term poor prognosis and those with longer life expectancies are distinct and clearly the risk of the underlying uncurable disease typically outweighs longer term concerns. For those with better prognosis, the concerns related to renal toxicity may be magnified.

Several strategies might be considered to overcome renal toxicity issues and salivary toxicity issues (52). Such issues include the use of larger molecules (such as antibodies) which do not reach the renal tubule or penetrate the salivary gland. In general the glomerulus filters molecules below the 30−50 kilodalton range. Interestingly the salivary binding seen with small PSMA binding molecules is not seen with antibodies. Thus, intact antibodies would not be expected to enter the renal tubule via the glomerulus or the salivary glands. Albumin-binding PSMA targeted molecules are postulated to have diminished renal excretion and less salivary uptake and several are in development as noted above. Renal dosimetry considerations are of significant concern to regulatory bodies such as the FDA. Salivary concerns have been raised by investigators and patients alike.

A potential issue with ^225^Ac is that multiple daughters are anticipated after the initial decay and some of these daughters, especially ^213^Bi have the potential to be excreted via the kidney. Isotopes with a single alpha emission would be expected to deliver radiation more specifically to the tumor. Thus isotopes such as ^212^Pb, ^149^Tb, or ^211^At might be considered “more targeted” than isotopes with multiple alpha emitting daughters (like ^225^Ac).

Several small molecular moieties have been postulated to be capable of selectively providing shielding isotopic damage. For instance a series of “Tris-POC” molecules have been shown to be capable of diminishing renal and salivary uptake of ^177^Lu-PSMA without altering tumor uptake (53). Other shielding agents are also in development. Mitigating concern about salivary and renal toxicities are of special interest in the clinical development of PSMA targeted alpha therapy.

Longer term issues with secondary cancers are also a concern in those with longer life expectancies. Radiation is a known carcinogen and the effects of radiation on subsequent cancer development is well-demonstrated from many angles. In the end there may be informed consents and accepted trade-offs in risk when it comes to treating cancer patients with agents known to harbor a carcinogenic risk. That said, many of the chemotherapies commonly used in oncology have similar risks and those therapies are part of the accepted armamentarium.

## Combination therapies with alphas and betas

Tandem data using combinations of alpha and beta emitters have been reported from a number of sites in retrospective studies. Given concerns with rate-limiting xerostomia and late renal toxicities especially with small molecules, ^225^Ac-PSMA agents at reduced doses could be of interest. Patients resistant to ^177^Lu-PSMA 617 have been treated with a tandem approach with provocative results. Several studies have been reported and a complete review is not warranted herein. As an example, in a study of 20 patients 6.9 GBq of ^177^LuPSMA-617 and 5.3 MBq of ^225^Ac-PSMA-617 were tested in the initial treatment cycle (54). A total of 65% of patients had PSA decline >50%. Xerostomia was reported as being more tolerable than the use of ^225^Ac-PSMA-617 alone (given the lower dose of the alpha emitter).

Preclinical data indicate that monoclonal (J591) PSMA binding may result in more sustained uptake of radiolabeled PSMA small molecules (^177^Lu-PSMA-617), and more cell kill (55). If true in the clinic, this would provide a strong rationale for combining a monoclonal with a radiolabeled therapeutic PSMA small molecule. The monoclonal antibody J591 chelated to ^225^Ac (^225^Ac-J591) will soon be being tested in tandem therapy with ^177^Lu-PSMA-I&T (NCT04886986).

## Developmental strategies for targeted alpha therapy

Prostate specific membrane antigen targeted alpha therapy appears quite active in every prostate cancer setting tested thus far. Debate ensues over the best patient to be treated, best inclusion/exclusion selection criteria, best molecule, best dose, best isotope, and best schedule. These debates are inevitable in the context of multiple small non-comparative studies, especially when financial conflicts of interest may be present. What is clear is that this class of agent needs to be properly tested in prospective trials with a regulatory focus. Selection of patients is key.

Should targeted alphas go after beta failure, or before betas, or in combination with betas? All studies are of interest but the path to regulatory approval might be fastest in the post-^177^Lu-PSMA space. What is the control group for a randomized trial? A “standard of care” control arm is typical and understanding the ethics and acceptability of the control group is a key consideration. Can the control group be an FDA approved beta emitter? That is reasonable given the degree of efficacy with alpha emitters noted in early trials. Inclusion and exclusion criteria can be debated, as can endpoints. Crossover designs are also important to consider. Though tumor shrinkage has been accepted as an endpoint in trials for accelerated approval, typical therapeutic trials in advanced prostate cancer have depended on overall survival in a controlled trial. Radiographic progression free survival using strict criteria proposed by the prostate cancer working group 3 is acceptable to the FDA (56). In addition, objective radiographic responses are of significant interest to the FDA. Definitions of radiographic progression free survival depends on traditional scans including bone scans and CT/MRI scans. PSMA-PET based endpoints and PSA declines are not acceptable at this time. Without regulatory approvals targeted alpha therapy will never reach the populations in need.

## Author contributions

OS reviewed extensively the literature and wrote the manuscript. AB involved with drafting and tables preparation for the manuscript. Both authors read and approved the final manuscript.
